# Standard opioid-containing versus opioid-sparing anesthesia on early postoperative recovery after video-assisted thoracic surgery: A propensity-weighted analysis

**DOI:** 10.3389/fsurg.2022.1015467

**Published:** 2022-10-21

**Authors:** Yan-yu Jiang, Zhen-ping Li, Ming Yao, Qing-he Zhou

**Affiliations:** ^1^ Graduate School, Bengbu Medical College, Bengbu, China; ^2^ Graduate School, Bengbu Medical College, Bengbu, China; ^3^ Department of Anesthesiology and Pain Medicine, The Affiliated Hospital of Jiaxing University, Jiaxing, China

**Keywords:** opioid-sparing anesthesia, video-assisted thoracic surgery, postoperative nausea and vomiting, inverse probability treatment weighting, postoperative recovery

## Abstract

**Purpose:**

Opioids have several adverse effects. At present, there are no large clinical studies on the effects of opioid-sparing anesthesia on early postoperative recovery after thoracoscopic surgery. This study was to investigate the effects of opioid-sparing anesthesia on early postoperative recovery after thoracoscopic surgery.

**Methods:**

Adult patients who underwent video-assisted thoracic surgery from 1 January 2019 to 28 February 2021 were enrolled by reviewing the electronic medical records. Participants were divided into opioid-sparing anesthesia (OSA group) and opioid-containing anesthesia (STD group) based on intraoperative opioid usage. The propensity-score analysis was to compare the early postoperative recovery of two groups. The outcome measurements included the incidence of postoperative nausea and vomiting (PONV) during an entire hospital stay, need for rescue antiemetic medication, postoperative-pain episodes within 48 h after surgery, need for rescue analgesia 48 h postoperatively, duration of postoperative hospital stay, length of PACU stay, postoperative fever, postoperative shivering, postoperative atrial fibrillation, postoperative pulmonary infection, postoperative hypoalbuminemia, postoperative hypoxemia, intraoperative blood loss, and intraoperative urine output.

**Results:**

A total of 1,975 patients were identified. No significant difference was observed in patient characteristics between the OSA and STD groups after adjusting for propensity score-based inverse probability treatment weighting. The incidence of postoperative nausea and vomiting was significantly lower in the OSA group than in the STD group (14.7% vs. 18.9%, *p *= 0.041). The rescue antiemetic use rate was lower in the OSA group than in the STD group (7.5% vs.12.2%; *p *= 0.002). PACU duration was longer in the OSA group than in the STD group (70.8 ± 29.0 min vs. 67.3 ± 22.7 min; *p* = 0.016). The incidence of postoperative fever was higher in the STD group than that in the OSA group (11.0% vs.7.7%; *p *= 0.032). There were no differences between the groups in terms of other outcomes.

**Conclusions:**

Our results suggest that opioid-sparing anesthesia has a lower incidence of postoperative complications than opioid-based anesthetic techniques.

## Introduction

Opioids are often used during the perioperative period for intraoperative analgesia and postoperative pain management. Since the 1960 s, opioids have become an indispensable component of modern anesthesia (1). Notwithstanding, the routine use of opioids during anesthesia has recently been interrogated. They have well-known short-term and potential long-term adverse effects on patients and society (2). Opioid-related adverse effects can manifest as a multitude of postoperative symptoms, ranging from postoperative nausea and vomiting (PONV), respiratory depression, delirium, and ileus to hyperalgesia (3). Hyperalgesia is a paradoxical phenomenon observed with sensitization of the nervous system that exacerbated painful responses to noxious stimulation (4). These adverse effects seem to accumulate as patients take opioids for longer periods after surgery (5).

Owing to the side effects of opioids, opioid-free anesthesia has recently attracted interest from anesthesiologists. In essence, opioid-free anesthesia is the practice of intraoperative anesthesia without opioid use (6). Several studies have investigated opioid-free anesthesia, which has proven feasible in many surgeries, including laparoscopic, gynecological, chest, and heart surgeries (7–9). Nonetheless, in a recent study, Beloeil et al. (10) noted that more patients under opioid-free anesthesia with dexmedetomidine experienced severe adverse events. Therefore, opioid-sparing anesthesia (OSA) is an alternative option. OSA achieves intraoperative analgesia by administering a minimal amount of opioid analgesics combined with non-opioid adjuvants during surgery (11). The current basis for reducing opioid use in the perioperative setting is a multimodal analgesia regimen comprising non-opioid anesthetics and regional anesthetic techniques (12). Using these methods, the anesthesiologist can take advantage of the multiple mechanisms of different pharmacological agents that potentially collaborate to achieve hypnosis, autonomic stability, weakening of sympathetic responses, and intraoperative and postoperative analgesia (13).

To date, no study has comprehensively reported OSA in video-assisted thoracic surgery. Moreover, in our hospital, a lot of thoracic surgeries have made use of OSA with dexmedetomidine. Hence, this study aimed to comparatively assess the effects of standard opioid-containing and opioid-sparing anesthetic techniques on early postoperative recovery after video-assisted thoracic surgery, using propensity-weighted analysis.

## Methods

### Study design and inclusion criteria

This single-center, retrospective study was approved by the Ethics Committee of the Affiliated Hospital of Jiaxing University (Jiaxing, China; No. 2021–410), which waived the requirement for written informed consent. The study enrolled adult patients consecutively who underwent video-assisted thoracic surgery from 1 January 2019 to 28 February 2021 by reviewing the electronic medical records at our hospital.

### Exclusion criteria

The exclusion criteria included (1) patients who had motion sickness, (2) patients who underwent other concurrent operations, (3) patients who underwent intraoperative thoracotomy conversion, and (4) patients taking opioids chronically prior to surgery.

### Grouping

All included patients were further categorized into two groups based on whether they continued the addition of opioids intraoperatively. In patients receiving OSA (OSA group), dexmedetomidine was used for intra-analgesia in patients in whom opioid addition was discontinued. In our study, we defined the group that continued receiving remifentanil during surgery as the opioid-based anesthetic technique group (STD group). All data were obtained from the Haitai electronic medical record system and Docare anesthesia clinical information system. Two investigators reviewed all data and conducted a consistency check after collection. Data were collected and analyzed privately with a specific identification number for each patient.

### Intraoperative and postoperative care

All patients underwent general anesthesia. Before anesthesia, patients were routinely monitored using electrocardiography, blood pressure measurement, and oxygen saturation monitoring. Patients underwent continuous radial artery puncture manometry before surgery if they had a normal Allen-test result. The Bispectral Index™ (BIS™) was used to adjust the depth of anesthesia to maintain the BIS between 40 and 60. The anesthesia program complied with the medication specifications of the anesthesiology department of the affiliated hospital of Jiaxing University. Based on ideal body weight, both groups of patients were induced to receive sufentanil or fentanyl (0.3–0.5 µg/kg or 3–5 µg/kg; Yichang Humanwell Pharmaceutical Co, Ltd, China), propofol 1–2.5 mg/kg, etomidate 0.15–0.3 mg/kg, cis-atracurium 0.15–0.2 mg/kg, or rocuronium bromide 0.6–1 mg/kg. Continuous inhalation of sevoflurane was administered after induction. Cis-atracurium was discontinuously added to maintain muscle relaxation as required. In the OSA group, the depths of anesthesia and analgesia were maintained using propofol (3–12 mg/kg/h) and dexmedetomidine (0.2–0.7 µg/kg/h). In the STD group, anesthesia was maintained using remifentanil (0.1–0.2 µg/kg/min; Yichang Humanwell Pharmaceutical Co, Ltd, China) and compounded with propofol (3–12 mg/kg/h). In both groups, the change in intraoperative anesthesia-related drug dosage was determined by the anesthesiologist in charge of the patient. In general, hemodynamic stability is maintained by adjusting the depth of anesthesia. In cases where the intraoperative mean arterial pressure was 30% lower than the baseline or systolic pressure <90 mmHg, an intravenous infusion of 6 mg ephedrine was rapidly administered. If the intraoperative mean arterial pressure was 30% higher than the baseline, 10 mg of urapidil was administered. During surgery, esmolol was used to treat tachycardia (heart rate >110 beats/min) during intubation and intraoperatively. If the patient’s heart rate was <50 beats/min or excessive salivary-gland secretion occurred, atropine (0.5 mg) was administered.

All patients were intubated with a double-lumen tube (Covidien LLC, China) using fiber-optic bronchoscope positioning and ventilated with the following parameter during one-lung ventilation: tidal volume of 6–8 ml/kg. The tracheal model was selected according to the height and diameter of the trachea in the chest computed tomography scan: 37 Fr/Ch (12.3 mm) was selected for men and 35 Fr/Ch (11.7 mm) for women. The ventilator parameters were adjusted to maintain the end-tidal carbon dioxide between 35 and 45 mmHg. During the procedure, all patients received 3 mg of granisetron or 5 mg tropisetron as preventative antiemetic medication. At the end of the surgery, non-steroidal anti-inflammatory drugs (NSAIDs) and opioid analgesics were administered for postoperative analgesia. NSAIDs used during surgery included flurbiprofen esters, ketorolac tromethamine, and parecoxib. Intraoperative opioid analgesics included dezocine (Yangtze River Pharmaceutical Co., Ltd., China) and butorphanol tartrate.

Patients in both groups underwent a systematic ultrasound-guided nerve block, including thoracic paravertebral block (TPVB, T_4–5_ level), serratus anterior paravertebral block (SAP, fourth and fifth rib level), pectoral nerves block (PECS, third and fourth rib level), erector spinae plane block(ESPB, T_4–5_ level) and epidural anesthesia (T_5–6_ level). A nerve block was generally performed by an anesthesiologist before the induction of anesthesia. The surgeon performed an intercostal nerve block after the operation if the anesthesiologist did not. Patients were immediately transferred to the post-anesthesia care unit (PACU) after operation and extubated in the PACU after neuromuscular blocking reversal. The pain was monitored using a numerical rating scale (NRS), rated from 0 to 10, and managed with tramadol titration if the pain was ≥3 in the PACU. In cases where the Aldrete score exceeded 9, patients were directly transferred to the ward (14). In the ward, patients received oxygen routinely for one day, and it was adjusted according to the patient’s condition. If the pain score was ≥3, the patients received NSAIDs or oxycodone orally. Patients were administered intramuscular morphine if the pain persisted or the NRS score was ≥6. Analgesic pumps were used according to the patients’ preferences. Intravenous analgesic pumps contain 100 µg sufentanil and 10 mg granisetron. The length of hospital stay was recorded from the end of the surgery to the time of discharge.

### Data collection

We reviewed the patients’ electronic medical records and collected their demographic characteristics, including age, sex, body mass index, and American Society of Anesthesiologists (ASA) physical status. We also collected data on potential risk factors that potentially affect outcomes, including coexistent disease (hypertension, diabetes, cardiovascular diseases, cerebrovascular disease, respiratory system diseases, and/or depression), surgery type (pulmonary wedge, pulmonary lobectomy, segmentectomy, pulmonary bulla resection, the number of trochal ports, mediastinal tumor resection, thymectomy, or thoracic sympathectomy), smoking status, anesthesia-induction drugs, anesthesia-maintenance drugs, other intraoperative drugs, perioperative nerve block, analgesic pump, surgical duration, duration of anesthesia, duration of mechanical ventilation, and amount of rehydration. These variables constituted the baseline indicators in our study.

### Outcomes

Endpoints that occurred during hospitalization were obtained from the inpatient electronic medical records. The primary outcome measure was the incidence of PONV during the entire hospital stay. We also investigated several other pre-specified secondary outcomes: the need for rescue antiemetic medication, postoperative-pain episodes (defined as any episode with an NRS >3) within 48 h after surgery, need for rescue analgesia 48 h postoperatively, duration of postoperative hospital stay, length of PACU stay, postoperative fever, postoperative shivering, postoperative atrial fibrillation, postoperative pulmonary infection, postoperative hypoalbuminemia, postoperative hypoxemia, intraoperative blood loss, and intraoperative urine output. The pain was measured using NRS scores, ranging from 0 to 10, and postoperative hypoxemia was defined as a SpO_2_ level <95% with a need for oxygen supplementation (15).

### Statistical analysis

Continuous variables are described as means ± standard deviations for normally distributed variables, medians and interquartile ranges [IQRs] for non-normally distributed variables, and frequencies and percentages for categorical variables. Between-group differences were reported using the independent samples *t-test*, chi-square (*χ*^2^) test, or nonparametric test, as appropriate, and the 95% confidence interval (CI) was calculated. Missing continuous variables were replaced by the median. In the current study, remifentanil administration was at the discretion of the attending anesthetist and was not randomly assigned to the subjects. To reduce bias in comparing non-randomized treatments, we calculated the propensity score for each subject. The propensity score is defined as the probability of treatment assignment conditional on the measured baseline covariates. In our study, the propensity score was the probability of receiving remifentanil and was estimated using a logistic regression model. To control for confounding factors in case of an unknown relationship between the groups (OSA and STD) and covariates, we used inverse probability treatment weighting (IPTW) using generalized boosted models or multivariate nonparametric regression techniques. IPTW weights were estimated as the inverse values of patients’ estimated probability of belonging to the OSA group. IPTW, based on propensity scores, was used to balance the distributions of the collected baseline variables in the OSA and STD groups (16). On applying IPTW, baseline variables were considered balanced between the OSA and STD groups if the absolute standardized mean difference was <0.1. All reported *p* values were two-sided, and values of *p* < 0.05 were considered statistically significant. All analyses were performed using R (version 4.1.2; the R Foundation for Statistical Computing, http://www.r-project.org).

## Results

### Patient characteristics

**Figure 1:**
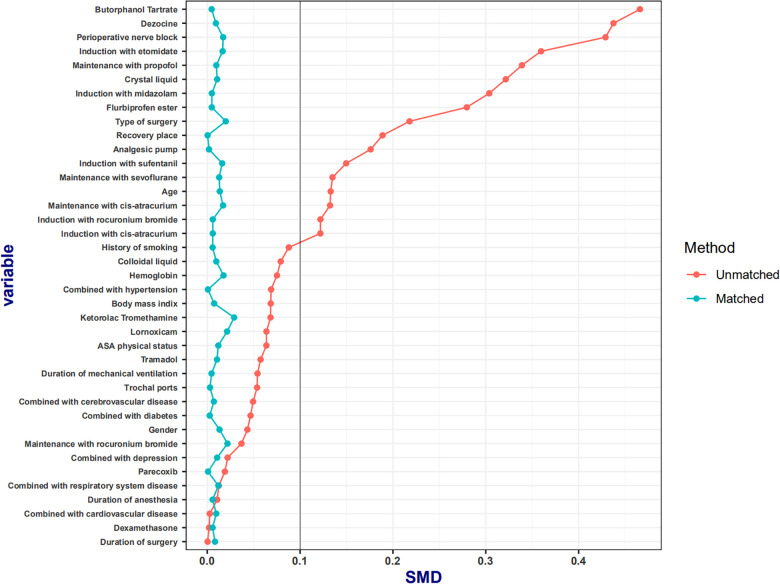
The absolute standardized mean differences in baseline characteristics between the two groups. The absolute standardized mean differences in all the baseline characteristics between the two groups were <10%.

A total of 1,975 patients were included in the present study (STD group, *n* = 681, 34.5%; OSA group, *n* = 1294, 65.5%). During induction, all patients received opioids comprising a sufentanil dose of 30 [30–30] μg (sufentanil and fentanyl are converted according to sufentanil: fentanyl = 10 μg: 0.1 mg, and all are expressed with sufentanil). In the STD group, remifentanil was administered at a dose of 450 [300–630] μg. In the OSA group, dexmedetomidine was administered at a dose of 40 [30–53] μg. Weighting by the inverse of the propensity score eliminated baseline-variable imbalances to produce a high degree of balance for these variables between the two groups. We compared baseline patient characteristics before and after weighting, and the results are shown in Table 1. Weighting reduced standardized differences for all observed covariates below 10% in absolute value, demonstrating substantial improvement in covariate balance across the treatment groups (Figure 1).

**Table 1:** Demographic and clinical characteristics of the two groups before and after propensity score weighted.

Variables	Before IPTW			After IPTW		
	Group STD, *n* = 681	Group OSA, *n* = 1294	*p-value*	Group STD, *n* = 489.86^a^	Group OSA, *n* = 489.57^a^	*p-value*
Age [mean (SD); years]	57.5 ± 13.2	55.7 ± 14.0	0.005	57.0 ± 13.3	56.8 ± 13.6	0.786
Male/female, no.	252/429	506/788	0.388	186.7 (38.1%)	190.2 (38.9%)	0.781
BMI [mean (SD); kg/㎡]	23.4 ± 3.1	23.2 ± 3.3	0.153	23.3 ± 3.1	23.3 ± 3.1	0.923
ASA, no. (%)			0.166			0.871
II	658 (96.6%)	1264 (97.7%)		471.9 (96.3%)	472.5 (96.5%)	
III	23 (3.4%)	30 (2.3%)		18.0 (3.7%)	17.1 (3.5%)	
History of smoking, no. (%)	58 (8.5%)	144 (11.1%)	0.081	44.2 (9.0%)	45.4 (9.3%)	0.880
Hemoglobin [mean (SD); g/L]	136.9 ± 15.2	138.0 ± 15.3	0.113	136.9 ± 15.3	137.1 ± 15.5	0.749
**Combined disease**						
Hypertension, no. (%)	220 (32.3%)	377 (29.1%)	0.159	149.2 (30.5%)	148.3 (30.3%)	0.943
Diabetes, no. (%)	62 (9.1%)	101 (7.8%)	0.362	42.6 (8.7%)	42.2 (8.6%)	0.965
Cerebrovascular disease, no. (%)	12 (1.7%)	32 (2.5%)	0.391	10.1 (2.1%)	9.6 (2.0%)	0.891
Cardiovascular diseases, no. (%)	27 (4.0%)	52 (4.0%)	1	21.1 (4.3%)	20.1 (4.1%)	0.857
Respiratory system diseases, no. (%)	10 (1.5%)	21 (1.6%)	0.943	7.2 (1.5%)	6.4 (1.3%)	0.794
Depression, no. (%)	9 (1.3%)	14 (1.1%)	0.802	6.0 (1.2%)	5.5 (1.1%)	0.873
Type of surgery			0.003			0.999
Pulmonary wedging, no. (%)	188 (27.6%)	403 (31.1%)		134.7 (27.5%)	134.0 (27.4%)	
Pulmonary lobectomy, no. (%)	220 (32.3%)	424 (32.8%)		162.7 (33.2%)	164.8 (33.7%)	
Pulmonary segmentectomy, no. (%)	218 (32.0%)	316 (24.4%)		146.4 (29.9%)	143.9 (29.4%)	
Pulmonary bulla resection, no. (%)	17 (2.4%)	58 (4.5%)		15.5 (3.2%)	15.6 (3.2%)	
Mediastinal tumor resection, no. (%)	21 (3.1%)	57 (4.4%)		18.0 (3.7%)	19.5 (4.0%)	
Thymectomy, no. (%)	17 (2.4%)	32 (2.5%)		12.5 (2.6%)	11.8 (2.4%)	
Thoracic sympathectomy, no. (%)	0 (0%)	4 (0.3%)		0.0 (0.0%)	0.0 (0.0%)	
**The number of VATS ports, no. (%)**						
Single port	315 (46.3%)	584 (43.6%)	0.277	224.5 (45.9%)	224.8 (46.0%)	0.957
Three ports	366 (53.7%)	710 (56.4%)		265.4 (54.1%)	264.8 (54.0%)	
**Intraoperative variables**						
Duration of surgery, (min)	90[65–120]	88.50[60–120]	0.402	97.1 ± 42.9	96.8 ± 43.6	0.883
Duration of anesthesia, (min)	110[90–145]	110[80–145]	0.490	118.6 ± 43.9	118.3 ± 45.1	0.913
Duration of mechanical ventilation, (min)	135[105–165]	135 [105–170]	0.690	140.8 ± 45.2	140.7 ± 45.9	0.936
Crystal liquid, (ml)	1000[500,1000]	500[500,1000]	<0.001	869.0 ± 369.7	872.5 ± 377.8	0.868
Colloidal fluid, (ml)	500[500,500]	500[500,500]	0.151	422.4 ± 245.8	419.6 ± 285.8	0.855
**Induction of anesthetics**						
Sufentanil (µg)^b^	30[25,30]	30[30,30]	0.702	29.1 ± 7.3	29.0 ± 5.3	0.760
Cis-atracurium, no. (%)	590 (86.6%)	1064 (82.2%)	0.014	418.0 (85.3%)	416.5 (85.1%)	0.892
Rocuronium bromide, no. (%)	91 (13.4%)	230 (17.8%)	0.014	71.8 (14.7%)	73.1 (14.9%)	0.892
Midazolam, no. (%)	123 (18.1%)	103 (8.0%)	<0.001	61.2 (12.5%)	60.9 (12.4%)	0.982
Etomidate, no. (%)	570 (83.7%)	888 (68.6%)	<0.001	388.7 (79.3%)	385.3 (78.7%)	0.767
**Maintenance anesthetics**						
Propofol, no. (%)	592 (86.9%)	1245 (96.2%)	<0.001	451.7 (92.2%)	450.2 (92.0%)	0.860
Sevoflurane, no. (%)	634 (93.8%)	1244 (96.3%)	0.004	457.9 (93.5%)	458.9 (93.7%)	0.845
Cis-atracurium, no. (%)	418 (61.4%)	710 (54.9%)	0.006	286.6 (58.5%)	282.2 (57.6%)	0.751
Rocuronium bromide, no. (%)	9 (1.3%)	23 (1.8%)	0.565	7.1 (1.4%)	8.5 (1.7%)	0.692
**Other intraoperative drugs**						
Dezocine, no. (%)	464 (68.1%)	1114 (86.1%)	<0.001	381.0 (77.8%)	378.7 (77.4%)	0.860
Ketorolac tromethamine, no. (%)	514 (75.5%)	938 (72.5%)	0.168	371.9 (75.9%)	364.6 (74.5%)	0.549
Parecoxib, no. (%)	15 (2.2%)	25 (1.9%)	0.812	13.2 (2.7%)	13.2 (2.7%)	1
Lornoxicam, no. (%)	7 (1.0%)	23 (1.8%)	0.271	7.0 (1.4%)	8.4 (1.7%)	0.691
Tramadol, no. (%)	1 (0.1%)	6 (0.5%)	0.467	1.0 (0.2%)	1.2 (0.3%)	0.857
Flurbiprofen ester, no. (%)	53 (7.8%)	24 (1.9%)	<0.001	22.3 (4.5%)	22.1 (4.5%)	0.982
Butorphanol tartrate, no. (%)	71 (10.4%)	3 (0.2%)	<0.001	2.8 (0.6%)	3.0 (0.6%)	0.919
Dexamethasone, no. (%)	192 (28.2%)	366 (28.3%)	1	140.7 (28.7%)	139.4 (28.5%)	0.924
Postoperative analgesic mode			0.001			1
PCIA, no. (%)	607 (89.1%)	1198 (92.6%)		451.7 (92.2%)	451.3 (92.2%)	
PCEA, no. (%)	2 (0.3%)	11 (0.9%)		2.0 (0.4%)	2.0 (0.4%)	
PCNA, no. (%)	3 (0.4%)	0 (0%)		0.0 (0.0%)	0.0 (0.0%)	
Perioperative nerve block			<0.001			1
Intercostal nerve blocks, no. (%)	591 (86.8%)	1258 (97.2%)		465.8 (95.1%)	464.8 (94.9%)	
SAP, no. (%)	31 (4.6%)	6 (0.5%)		6.6 (1.3%)	6.0 (1.2%)	
TPVB, no. (%)	34 (5.0%)	10 (0.8%)		7.1 (1.4%)	7.9 (1.6%)	
PECS, no. (%)	12 (1.8%)	4 (0.3%)		3.7 (0.8%)	4.0 (0.8%)	
ESPB, no. (%)	10 (1.5%)	5 (0.4%)		4.8 (1.0%)	4.9 (1.0%)	
Epidural anesthesia, no. (%)	3 (0.4%)	11 (0.9%)		2.0 (0.4%)	2.0 (0.4%)	

^a^ The number of subjects changed after IPTW in the calculation; however, the actual number of subjects did not change.

^b^ Sufentanil and fentanyl are converted according to sufentanil: fentanyl = 10µg: 0.1 mg, and all are expressed with sufentanil; cerebrovascular disease including cerebral infarction; cardiovascular diseases including coronary heart disease, arrhythmia, heart failure; respiratory system diseases including chronic obstructive pulmonary disease, asthma, bronchitis, bronchiectasis.

The data are presented as means ± SD for continuous variables and frequency (%) for categorical variables. IPTW, inverse probability of treatment weighting; BMI, body mass index; ASA, American Society of Anesthesiologists; PCEA, patient controlled epidural analgesia; PCIA, patient controlled intravenous analgesia; PCNA, patient controlled nerve analgesia; SAP, serratus anterior plane block; TPVB, thoracic paravertebral block; PECS, pectoral nerves block; ESPB, Erector spinae plane block.

### Postoperative outcomes after weighting

Approximately 16.5% (326/1975) of patients developed PONV during hospitalization. The PONV rate was significantly lower in the OSA group than in the STD group after weighting (14.7% *vs.*18.9%; *p* = 0.041), and there was a significant decrease in the use of rescue antiemetics in the OSA group (7.5% vs. 12.2%; *p* = 0.002). PACU duration was longer in the OSA group than in the STD group (70.8 ± 29.0 vs. 67.3 ± 22.7; *p* = 0.016). The incidence of postoperative fever was higher in the STD group than that in the OSA group (11.0% vs. 7.7%; *p *= 0.032). There were no differences in the number of cases with NRS scores >3, hospital length of stay, and postoperative rescue analgesia. No differences were found in the incidence of postoperative urinary retention, postoperative shivering, postoperative atrial fibrillation, postoperative pulmonary infection, postoperative hypoalbuminemia, and postoperative hypoxemia between the two groups (Table 2).

**Table 2:** Outcome analyses.

Outcome	Before IPTW			After IPTW		
	Group STD, *n* = 681	Group OSA, *n* = 1294	*p-value*	Group STD, *n* = 489.86^a^	Group OSA, *n* = 489.57^a^	*p-value*
PONV, no. (%)	132 (19.4%)	196 (15.1%)	0.019	92.5 (18.9%)	72.2 (14.7%)	0.041
Use of rescue antiemetic, no. (%)	87 (12.8%)	101 (7.8%)	<0.001	59.9 (12.2%)	36.5 (7.5%)	0.002
Postoperative urinary retention, no. (%)	5 (0.7%)	7 (0.5%)	0.825	4.6 (0.9%)	2.5 (0.5%)	0.361
Postoperative fever, no. (%)	74 (10.9%)	108 (8.3%)	0.079	53.9 (11.0%)	37.5 (7.7%)	0.032
Postoperative shiver, no. (%)	12 (1.8%)	9 (0.7%)	0.049	7.0 (1.4%)	5.2 (1.1%)	0.544
Postoperative atrial fibrillation, no. (%)	28 (4.1%)	70 (5.4%)	0.249	19.1 (3.9%)	29.6 (6.1%)	0.083
Postoperative pulmonary infection, no. (%)	82 (12.0%)	205 (15.8%)	0.027	61.6 (12.6%)	79.9 (16.3%)	0.058
Postoperative hypoalbuminemia, no. (%)	11 (1.6%)	19 (1.5%)	0.952	8.5 (1.7%)	8.7 (1.8%)	0.958
postoperative hypoxemia, no. (%)	22 (3.2%)	32 (2.5%)	0.403	13.8 (2.8%)	12.4 (2.5%)	0.744
Number of episodes with NRS > 3, no. (%)	42 (6.2%)	55 (4.3%)	0.078	27.7 (5.7%)	23.5 (4.8%)	0.496
Hospital LOS, (day)	5.9 ± 3.6	6.1 ± 3.7	0.297	6.0 ± 3.7	6.2 ± 3.5	0.371
PACU LOS, (min)	67.1 ± 22.2	72.2 ± 28.9	<0.001	67.3 ± 22.7	70.8 ± 29.0	0.016
Intraoperative blood loss, (ml)	46.3 ± 99.3	50.6 ± 110.8	0.401	45.7 ± 85.5	55.9 ± 132.1	0.086
Intraoperative urine output, (ml)	213.5 ± 192.1	216.6 ± 182.7	0.731	222.5 ± 204.6	218.8 ± 180.3	0.736
**Postoperative rescue analgesia in 48 h**						
Morphine, no. (%)	18 (2.6%)	15 (1.2%)	0.024	10.7 (2.2%)	8.0 (1.6%)	0.476
Oxycodone, no. (%)	8 (1.2%)	25 (1.9%)	0.288	5.9 (1.2%)	7.7 (1.6%)	0.572
NSAIDs, no. (%)	32 (4.7%)	52 (4.0%)	0.552	24.1 (4.9%)	19.0 (3.9%)	0.348

^a^ The number of subjects changed after IPTW in the calculation; however, the actual number of subjects did not change. Categorical variables data are presented as median (25th percentile-75th percentile) and frequency (%). PONV, postoperative nausea and vomiting; NRS, numerical rate scale; LOS, length of stay; PACU, post-anesthesia care unit; NSAIDs, nonsteroidal anti-inflammatory drugs.

### Operative outcomes after weighting

Intraoperative blood loss and urine output did not differ between the STD and OSA groups (*p *> 0.05) (Table 2). Intraoperative atropine use was lower in the STD group (*p *< 0.05); however, there was no significant difference in the intraoperative use of esmolol, ephedrine, and norepinephrine between the two groups (*p *> 0.05) (Table 3).

**Table 3:** Using of vasoactive drugs during operation.

Outcome	Before IPTW			After IPTW		
	Group STD, *n* = 681	Group OSA, *n* = 1294	*p-value*	Group STD, *n* = 489.86^a^	Group OSA, *n* = 489.57^a^	*p-value*
Ephedrine, no. (%)	56 (8.2%)	140 (10.8%)	0.079	41.9 (8.6%)	52.2 (10.7%)	0.200
Deoxyepinephrine, no. (%)	154 (22.6%)	203 (15.7%)	<0.001	98.1 (20.0%)	93.8 (19.2%)	0.690
Atropine, no. (%)	295 (43.3%)	371 (28.7%)	<0.001	187.6 (38.3%)	147.7 (30.2%)	0.002
Esmolol, no. (%)	303 (44.5%)	491 (37.9%)	0.006	179.5 (36.7%)	177.9 (36.3%)	0.908

^a^ The number of subjects changed after IPTW in the calculation; however, the actual number of subjects did not change.

## Discussion

In this retrospective study, we compared the effects of standard opioid-containing anesthesia with those of OSA on postoperative recovery after video-assisted thoracic surgery using propensity score-based IPTW. Our findings demonstrated that OSA was associated with a decrease in PONV, a reduction in postoperative remedial antiemetic incidence, and a decrease in postoperative fever, while the duration of PACU stays in patients under OSA was prolonged.

A large number of patients experience PONV after general anesthesia (17–19). The application of opioids in the perioperative period is one of the major reasons for this phenomenon (20–22). Previous studies have reported conflicting results regarding the effect of remifentanil on PONV. In a small randomized controlled trial, Watanabe et al. (23) reported that remifentanil was rapidly excreted and did not affect the incidence of PONV. However, a retrospective study performed on 423 patients who underwent elective mastectomy under general anesthesia reported the incidence of PONV with remifentanil to be similar to that with other opioids and may be highly correlated with the dose of remifentanil (24). In our study, patients who underwent OSA exhibited a lower incidence of PONV, and the frequency of postoperative rescue-antiemetic use was also significantly reduced. The correlation between the intraoperative dosage of remifentanil and PONV warrants further confirmation by a well-designed, prospective, large-sample trial.

The difference in PACU duration between the two groups was notable yet not surprising. In the current study, patients with opioid-free anesthesia increased the use of dexmedetomidine. Although many factors affect the duration of PACU, in this study, we think that the main reason for the longer PACU duration is that the half-life of dexmedetomidine is significantly longer than that of remifentanil (25). Recently, Devine et al. (26) conducted a case-control study to investigate opioid-free anesthesia for lung cancer resection. In that study, PACU duration in patients under opioid-free anesthesia was also longer than that under standard opioid-containing anesthesia, which is consistent with our study’s results. Arguably, an OSA technique embracing a multimodal analgesic regimen may contribute to reductions in PONV and, if present, potentially prolong PACU duration to a certain extent.

There were no differences in postoperative pain scores and use of rescue analgesia within 48 h after surgery. Several studies have suggested that the intraoperative pumping of remifentanil increases the use of postoperative analgesics (10, 27). High-dose remifentanil use was associated with higher postoperative pain scores and opioid consumption. This phenomenon is most likely due to the development of acute opioid tolerance (28). In the current study, we combined other analgesics, such as NSAIDs and sevoflurane, with remifentanil; thus, the dosage of remifentanil was less than that in the abovementioned report (10).

Perhaps the unexpected finding of the current study was that patients under OSA exhibited a lower probability of fever than those under standard opioid-containing anesthesia. Dexmedetomidine reduces central sympathetic nerve activity, which potentially inhibits of the production of proinflammatory cytokine (29, 30). It is likely that the anti-inflammatory effect of dexmedetomidine potentially benefits patients with fever (31). However, no difference in postoperative lung infection was found between the two groups. The cause of lung infection is multifactorial and may be influenced by other factors, such as the external environment and care factors. Other outcomes, such as postoperative hypoxemia, postoperative urinary retention, and length of hospital stay, did not differ significantly between the two groups.

In the present study, we also compared the application of intraoperative vasoactive drugs and found that patients administered standard opioid-containing drugs were more likely to use atropine than patients administered OSA. Dexmedetomidine, a selective α2-adrenoceptor agonist, tends to lower heart rate (32). A previous study demonstrated that heart rate decreased transiently soon after the initiation of remifentanil infusion (33). Considering that patients were in a lateral position during surgery, the surgical use of atropine is not exclusively for the purpose of raising heart rate but also to reduce oral secretion (34). Thus, the difference in atropine use between the two groups may be related to reducing oral secretion. Differences in the use of ephedrine, esmolol, and deoxygenation were not statistically significant between the two groups, indicating that the hemodynamic fluctuations of the two groups were similar.

Our study has certain limitations. First, because of the retrospective nature of the study, undiscovered confounding factors might have remained, despite the use of propensity-score analytics. However, such confounders would need to be very significant to invalidate the results of this study. Second, due to the large number of baseline indicators included in the present study, many baseline indicators, such as auxiliary drugs used in anesthesia, were considered categorical variables, and no quantitative analysis was performed. Third, the results revealed that OSA was associated with a decreasing PONV incidence. PONV was not recorded in the medical record system according to a four-point scale (35). Therefore, we could not analyze the severity of postoperative nausea and vomiting in both patient groups.

## Conclusions

We found OSA to be associated with decreases in PONV, postoperative remedial antiemetic use, and postoperative fever incidence after video-assisted thoracic surgery. However, the findings of our study, which were based on a non-randomized design, warrant similar analyses using larger sample sizes, prospective follow-up studies, and confirmation in randomized clinical trials.

## Data Availability

The raw data supporting the conclusions of this article will be made available by the authors, without undue reservation.
